# Interferon-γ and Interleukin-10 Profiles Correlate with Disease Severity in Sudanese Children with *Plasmodium falciparum* Malaria

**DOI:** 10.3390/jcm15103929

**Published:** 2026-05-20

**Authors:** Tassneem Awad Hajali, Muna Ismail Elmisbah Mekki, Nouralsalhin A. Alaagib, Islamia Ibrahim Ahmed Omer, Rabab Mahadi Yahia, Hamdan Z. Hamdan

**Affiliations:** 1 Department of Clinical Science, College of Medicine, Sulaiman AlRajhi University, AlBukayriah 51941, Qassim, Saudi Arabia; t.hajali@sr.edu.sa (T.A.H.);; 2 Department of Pediatrics, College of Medicine, Sudan University for Science and Technology, Khartoum 11111, Sudan; 3 Department of Basic Science, College of Medicine, Sulaiman AlRajhi University, AlBukayriyah 51941, Qassim, Saudi Arabia; 4 Department of Pediatrics, Maternity and Children Hospital, Buraidah 52384, Qassim, Saudi Arabia; 5 Department of Pathology, College of Medicine, Qassim University, Buraidah 51452, Qassim, Saudi Arabia

**Keywords:** *Plasmodium falciparum*, severe malaria, interferon-gamma, interleukin 10, cytokines, biomarkers, Sudanese children

## Abstract

**Background/Objectives**: Severe *Plasmodium falciparum* malaria remains a leading cause of childhood morbidity and mortality in sub-Saharan Africa. The clinical outcome is largely driven by the balance between pro-inflammatory and anti-inflammatory cytokines. However, immunological data from Sudanese children remain limited and the diagnostic utility of cytokine biomarkers has not been formally evaluated in this population. Therefore, this study aims to compare plasma levels of IFN-γ and IL-10 among Sudanese children with severe malaria (SM), uncomplicated malaria (UM) and healthy controls (HC) and to evaluate their diagnostic accuracy using in differentiating SM from UM cases. **Methods**: A hospital-based case–control study was conducted at Mohamed Elamin Hamid Pediatric Hospital, Omdurman, Sudan. The study enrolled 105 children (aged 3 months to 16 years) divided into three age- and sex-matched groups (n = 35 per group): SM, UM and HC. Plasma cytokines IFN-γ and IL-10 were measured by ELISA. **Results**: The anthropometric measurements did not differ significantly across the groups. SM was significantly associated with lower hemoglobin and markedly reduced platelet counts. Both IFN-γ and IL-10 were significantly elevated in SM versus UM and HC (*p* < 0.001). A strong positive correlation was observed between IFN-γ and IL-10 (rho = 0.688, *p* < 0.001) in malaria patients. Additionally, a significant correlation was observed between IL-10 and platelet count (rho = −0.338, *p* = 0.009). Both IL-10 (AUC = 0.0.720) and IFN-γ (AUC = 0.687) demonstrated moderate diagnostic performance in discriminating between SM vs. UM. **Conclusions**: Elevated levels of IFN-γ and IL-10 strongly associated with severe *P. falciparum* malaria in Sudanese children. Measuring IL-10 and IFN-γ at the time of presentation may differentiate between SM and UM cases. Further studies are needed.

## 1. Introduction

Severe *Plasmodium falciparum* (*P. falciparum*) malaria continues to be a significant cause of morbidity and mortality among children worldwide. Globally, around 282 million malaria cases and 608,000 deaths are reported annually, with nearly 95% of cases and deaths occurring among African children [1,2,3,4]. The sub-Saharan Africa region bears the highest burden, accounting for 76% of malaria deaths on the continent and 96% globally. Sudan is not an exception; malaria continues to be a massive public health challenge there, with 3,406,260 reported cases and 82,559 deaths among children under five were reported in 2022 [5], contributing to a large proportion of pediatric hospital admissions and a considerable number of deaths among children admitted to intensive care units [6,7].

The clinical presentation of *P. falciparum* malaria has a broad spectrum [8]. It ranges from asymptomatic parasitemia and uncomplicated malaria (UM) to severe, life-threatening malaria forms (SM) characterized by severe presentation such as cerebral malaria, severe anemia, and acute kidney injury [7,9,10]. These variable clinical presentations are largely shaped by the host’s immune system, in which cytokine interactions play a major regulatory role [11,12]. A successful immune defense is thought to rely on a coordinated regulatory loop: pro-inflammatory cytokines first work to control the initial parasite load, followed by anti-inflammatory mediators that limit excessive host tissue damage and prevent severe immunopathology [13]. Disruption of this homeostatic balance is considered a cornerstone in the pathogenesis of SM. Elevated levels of pro-inflammatory mediators, including interferon-gamma (IFN-γ), have been consistently associated with severe disease manifestations, microvascular obstruction, and organ dysfunction [14,15,16]. Conversely, the anti-inflammatory cytokine interleukin-10 (IL-10) is typically upregulated to counteract this inflammatory burst; however, paradoxically high levels of IL-10 have also been observed with increased disease severity, higher parasite densities, and poor prognosis in pediatric populations [14,17,18,19], which creates an ambiguity regarding the exact role of cytokines in severe malarial infection in children.

Based on the current literature, a limited number of studies have investigated the cytokine circulatory signature in Sudanese children [17,20,21]. To date, no studies have examined the cytokine profile among pediatric malaria patients in the capital region. Additionally, the diagnostic potential of these cytokines in differentiating severe from uncomplicated cases were lacking in Sudan. Therefore, the current study aims to investigate the cytokine circulatory profile in pediatric patients with severe malaria and to correlate cytokine levels with the clinical and laboratory indicators of disease severity, and to evaluate their diagnostic ability in differentiating SM from UM cases. Therefore, this study will add to the Sudanese and African literature.

## 2. Materials and Methods

### 2.1. Study Design and Setting

A hospital-based case–control study was conducted at the Mohamed Elamin Hamid Pediatric Hospital in Omdurman, Sudan. The hospital serves a large catchment area in Omdurman and surrounding communities, where malaria transmission is seasonal.

### 2.2. Study Population and Case Definitions

A total of 105 Sudanese children aged 3 months to 16 years were enrolled in this study. Participants were divided into three groups matched by age and sex (n = 35 per group): severe malaria (SM), uncomplicated malaria (UM) and a healthy control group (HC). Malaria cases were diagnosed and classified according to the WHO criteria for malaria severity [22]. The SM group included children admitted with microscopically confirmed *P. falciparum* parasitemia accompanied by one or more severe manifestations such as severe malarial anemia, cerebral malaria, prostration, hypoglycemia or repeated convulsions. The UM group consisted of febrile children with symptomatic *P. falciparum* infection but without any signs of severe disease. The HC group comprised apparently healthy, afebrile children attending outpatient clinics for routine check-ups or vaccinations and who had negative Giemsa-stained blood smears for malaria.

Children were excluded if they were non-Sudanese, were younger than 3 months of age, had concurrent infectious diseases or severe malnutrition, or were receiving immunosuppressive therapy. Additional exclusion criteria included the presence of other febrile illnesses, a known chronic disease, or a lack of written informed consent from parents or legal guardians.

### 2.3. Data Collection, Clinical Assessment, and Laboratory Procedures

Upon admission, all participants underwent a standard clinical examination and anthropometric measurements, including weight, height, and head circumference. A structured questionnaire was used to collect socio-demographic, clinical and laboratory data, including age, gender, anthropometric measurements, clinical symptoms, and laboratory results such as, CBC, blood glucose, urea levels, and cytokine measurements. After obtaining informed consent from the parents, 5 mls of venous blood was collected under strict aseptic conditions and kept in appropriate vacutainer tubes for routine laboratory investigations and cytokine measurements.

Thick and thin Giemsa-stained blood films were prepared and examined microscopically to confirm parasitemia and quantify parasite density. Routine laboratory investigations, including complete blood counts (CBC), random blood glucose, blood urea, serum creatinine, and urine analysis, were performed using a fully automated clinical chemistry analyzer according to the manufacturer’s instructions.

### 2.4. Cytokine Measurement

Plasma was harvested after centrifugation at 3000 rpm for 10 min, kept in a sterile Eppendorf tube and then stored at −80 °C degree until completion of sample collection. The quantitative measurement of plasma IFN-γ and IL-10 concentrations was done using commercially available sandwich Enzyme-Linked Immunosorbent Assay (ELISA) kits (R&D Systems, Minneapolis, MN, USA) according to the manufacturer instructions. Optical densities were read at 450 nm using a microplate ELISA reader, and cytokine concentrations were determined based on standard curves.

### 2.5. Statistical Analysis

Data were analyzed using SPSS version 26.0 (IBM Corp., Armonk, NY, USA, 2019). Following normality assessment via the Shapiro–Wilk test, non-normally distributed continuous variables were presented as medians with interquartile ranges (IQRs) and compared using the Kruskal–Wallis test followed by the Mann–Whitney U test. Categorical variables were compared using the Chi-square or Fisher’s exact tests. To evaluate correlations between cytokines and clinical parameters, Spearman rank correlations (r) were utilized. The diagnostic performance of individual biomarkers was evaluated using Receiver Operating Characteristic (ROC) curve analysis to calculate the Area Under the Curve (AUC), sensitivity and specificity at the optimal Youden Index. A two-tailed *p*-value < 0.05 was considered statistically significant across all tests.

## 3. Results

**Demographic, Anthropometric, and Clinical Characteristics**

A total of 105 children were enrolled in the study, comprising 35 with SM, 35 with UM and 35 in the HC group. Age and sex distributions were comparable across the three groups, with no statistically significant differences in age (*p* = 0.730) (see Table 1). The median (inter-quartile range) for age was 5.0 (2.0–10.0) years in the SM group and 6.0 (2.0–9.8) years in UM and 6.0 (3.5–10.5) years in the HC group, with males representing 51.4% in each group.

There was no significant difference in weight and height among the SM, UM, and HC groups (*p* = 0.231 and *p* = 0.219, respectively). Head circumference was significantly lower in children with SM (median 49.0 cm) compared with those UM (52.0 cm) and HC (53.0 cm; *p* = 0.006). Children with SM had a significantly longer hospital stay 2.0 (2.0–3.0) vs. 1.0 (1.0–1.0) days in UM; *p* < 0.001).

**Hematological and biochemical profiles**

The median (interquartile) for hemoglobin concentration was significantly lower in the SM group 10.5 g/dL, (7.5–11.6) than in the UM 11.7 g/dL (11.2–12.2) and HC groups 11.9 g/dL (11.3–12.9); *p* = 0.001). Platelet counts were markedly reduced in the SM group [median 85.0 × 10^3^/μL, IQR (46.5–120.0)] compared with the UM (205.5 × 10^3^/μL) and HC (290.0 × 10^3^/μL; *p* < 0.001). White blood cell counts did not differ significantly among the groups (*p* = 0.302) (see Table 2). No statistically significant differences were observed among the SM, UM, and HC groups regarding median random blood glucose, blood urea and serum creatinine values (see Table 2).

**Plasma cytokine levels**

**Figure 1:**
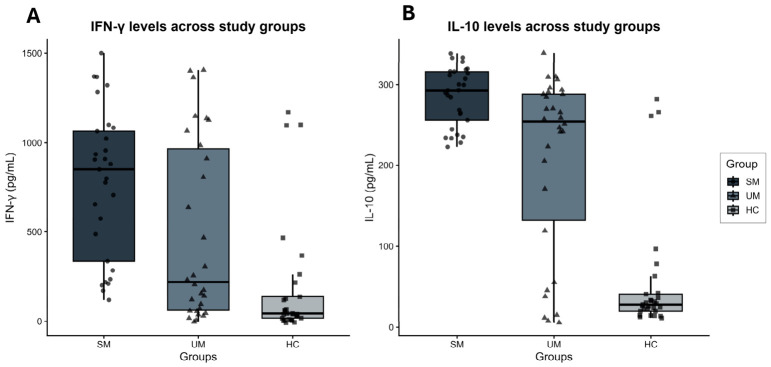
Plasma levels of (**A**) interferon-gamma (IFN-γ) and (**B**) interleukin-10 (IL-10) in the study groups. The horizontal line inside the box represents the median, while the box edges represent the interquartile range (IQR: 25th–75th percentiles).

Median (IQR) levels of IFN-γ concentrations were highest in children with SM [848.8 (335.4–1064.9); pg/mL], intermediate in UM [219.0 (61.6–966.6); pg/mL] and lowest in HC [43.6 (17.7–137.8), pg/mL], overall *p* < 0.001 (see Figure 1). Post hoc pairwise analysis demonstrated significantly higher IFN-γ levels between SM and UM (*p* = 0.014) and between SM and HC (*p* < 0.001). UM also differed significantly from HC (*p* = 0.004). A similar pattern was observed for IL-10; median IL-10 levels were highest in SM [292.4 (256.1–315.8); pg/mL] and UM [254.4 (1.9–288.1); pg/mL], both significantly elevated compared with HC [28.1 (19.9–41.1); pg/mL], overall *p* < 0.001). Severe malaria cases had significantly higher IL-10 concentrations than both UM (*p* = 0.004) and HC (*p* < 0.001), see Table 2.

**Clinical manifestations and laboratory abnormalities**

All children in both malaria groups presented with fever, and vomiting was common in SM (100%) and UM (91.4%), without a significant difference between the groups. In contrast, signs of severity were observed exclusively in the SM group, as shown in Table 3.

Thrombocytopenia affected 82.9% of the SM children compared with 31.4% of the UM children (*p* < 0.001). Additionally, severe anemia was significantly more common in SM (42.9% vs. 5.7%, *p* < 0.001), and hypoglycemia occurred in 20.0% of SM versus 5.7% of UM cases (*p* = 0.046. No significant differences were detected between the groups in the proportions of children with elevated blood urea, raised serum creatinine, leukocytosis, or leukopenia.

**Correlations between cytokines and clinical/laboratory parameters**

**Figure 2:**
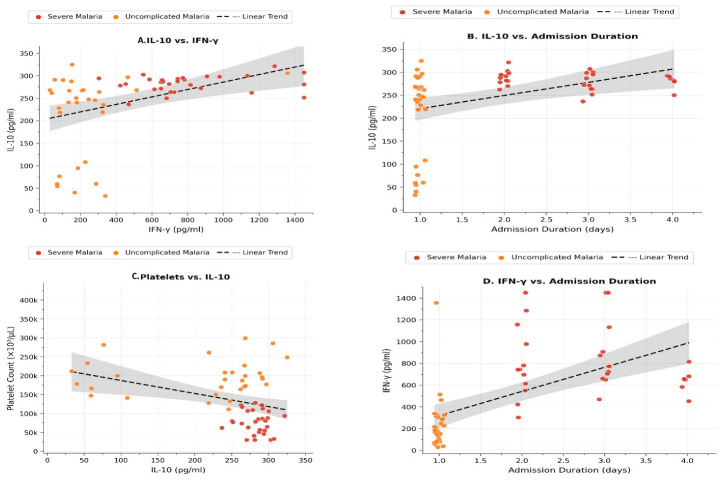
Scatter plots illustrate the correlations between serum cytokines (IL-10, IFN-γ) and key clinical/laboratory parameters in malaria patients: (**A**) Strong positive correlation between IL-10 and IFN-γ; (**B**) Positive association between admission duration and IL-10 levels; (**C**) Inverse correlation between platelet count and IL-10 levels; (**D**) Positive association between admission duration and IFN-γ.

Spearman’s correlation analysis among all malaria patients (SM and UM) showed a strong, highly significant positive association between IL-10 and IFN-γ (rho = 0.688, *p* < 0.001) (see Figure 2A). Also, IL-10 levels correlated positively with the duration of hospital admission (rho = 0.314, *p* = 0.015), (see Figure 2B), and inversely with the platelet count (rho = −0.338, *p* = 0.009), Figure 2C. IFN-γ showed a similar positive correlation with admission duration (rho = 0.298, *p* = 0.022), (see Figure 2D), while its negative correlation with the platelet count did not reach statistical significance (rho = −0.213, *p* = 0.105). No significant correlations were observed between either cytokine and other variables, including hemoglobin, white blood cell count, blood urea, or serum creatinine (see Table 4). All children survived and were discharged after the course of admission in good clinical condition.

**ROC Curve Analysis and Diagnostic Performance**

**Figure 3:**
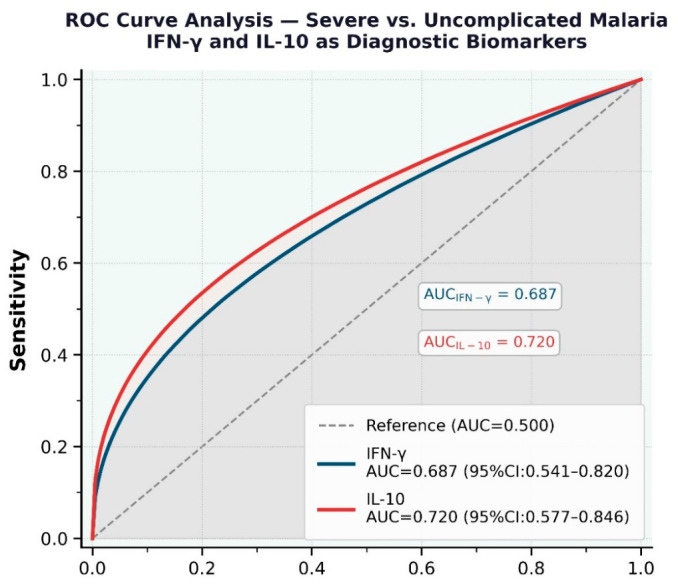
Receiver Operating Characteristic (ROC) curves for IFN-γ and IL-10 models. Severe vs. Uncomplicated Malaria (n = 35 per group). Dashed diagonal = reference line (AUC = 0.500). Notes: Bootstrap CI: 2000 iterations. Cutoff by Youden Index.

ROC analysis evaluated the diagnostic accuracy of IFN-γ and IL-10 in distinguishing SM from UM. Both cytokines showed moderate performance: IFN-γ had an AUC = 0.687 (95%CI: 0.541–0.820) and IL-10 had an AUC = 0.720 (95%CI: 0.580–0.845), with a sensitivity of 96.6% for IFN-γ and 100.0% for IL-10, and a specificity of 43.3% for IFN-γ and 33.3% for IL-10 (see Figure 3).

## 4. Discussion

The core finding in the current study is that both IFN-γ and IL-10 are significantly and simultaneously elevated in children with SM. Our findings are consistent with a previously reported study from Sudan [20] and are in agreement with findings from other African cohorts in Mali, Malawi and Togo [14,18,23,24]. Interferon-gamma, as a key pro-inflammatory cytokine, is primarily produced by Th1 cells and plays an important role in limiting the progression of uncomplicated malaria toward SM [25]. It is widely accepted that IFN-γ is acting against *P. falciparum* parasitemia [26]; however, its biological action is not limited to hindering parasite invasion. It induces a state of systemic inflammation by inducing other cytokines, namely TNF-α, IL-1β, IL-6 and others, which can amplify the inflammation further [27,28]. This systemic inflammation manifests as increased expression of endothelial adhesion molecules, which in turn cause sequestration of the parasitized erythrocyte in the microcirculation [27]. Such sequestration is responsible for microvascular obstruction, impaired tissue perfusion, and organ dysfunction, which are the hallmarks of severe malaria [29]. On the other hand, IL-10 acts as an anti-inflammatory cytokine that aims to ameliorate the toxicity of the pro-inflammatory cytokines. However, this neutralization is thought to be associated with a decreased the ability to clear *P. falciparum* parasitemia [30]. This is particularly true for *P. falciparum*, as it turns out that the IL-10 downregulates the expression of CD44 [31], the receptor used by *P. falciparum* to gain access into tissues, mainly the brain and lungs [32]. By doing so the parasite will be confined to the circulation and forced to face the different members of cellular and humoral immunity. Yet, this also may end in either SM form or a chronic inflammation state [30]. In this study, we observed a strong correlation between both IFN-γ and IL-10. This simultaneous elevation of opposing cytokines indicates an intense and dysregulated immune activation, which manifest as SM form.

In this study, we evaluated the diagnostic potential of IL-10 and IFN-γ as biomarkers for differentiating severe malaria from uncomplicated malaria at the time of clinical presentation. Early discrimination between these clinical forms is particularly valuable because it may help clinicians prioritize cases that require highly specialized care units, whereas those with less severe manifestations may be managed in a general ward or, when appropriate, through outpatient care [33]. However, a limited number of studies have investigated the diagnostic performance of cytokines in the background of severe malaria in children. A Ghanian cohort study assessed a panel of cytokines, including IL-10 and IFN-γ, in pediatric patients with severe and uncomplicated malaria and they found that only IL-17A (AUC = 82.4%) and IL-1β (AUC = 72.7%) showed excellent and moderate discriminatory performance, respectively, in differentiating SM from UC controls [34]. In the current study, IL-10 showed an AUC of 72.0%, which is exactly the performance of IL-1β reported in the Ghanian cohort, while IFN-γ showed an AUC of 68.7%, approaching 70.0%, the threshold typically considered indicative of low-to-moderate performance. Perhaps, integrating more cytokine measurements with clinical assessment at the time of presentation may enhance early case stratification and improve case management.

Another finding in the current study is the inverse correlation between IL-10 levels and platelet count. Our findings are consistent with the findings of Casals-Pascual et al. [35]. IL-10 is an anti-inflammatory cytokine that is induced to counteract the activity of pro-inflammatory cytokines such as IFN-γ and TNF-α. These pro-inflammatory cytokines promote the expression of endothelial adhesion molecules, such as ICAM-1, VCAM-1 and E-selectin, which enhance the adhesion of platelets and parasitized erythrocytes to the vascular endothelium [36]. Moreover, platelets have been shown to exert direct antiparasitic activity by targeting parasitized erythrocytes. This interaction consequently leads to platelet aggregation and consumption, thereby contributing to thrombocytopenia [37]. Furthermore, pro-inflammatory cytokines induce macrophages in the liver and spleen to phagocyte and clear the clumped platelets with infected erythrocytes, which further accelerate the platelet clearance and reduction in platelet counts [38]. Interestingly, Sosman and colleagues claimed that introducing IL-10 to healthy volunteers induced thrombocytopenia, which suggests that IL-10 may have a direct role in thrombocytopenia [39]. The exact mechanism remains poorly understood; yet, it has been proposed that IL-10 may suppress the production of megakaryocyte colony-forming unit [39]. Perhaps IL-10 impacted the megakaryocyte division/differentiaon and this is reflected directly in the patients. We can understand the direct effect of IL-10 on platelet counts in the context of mitigating disease severity. Fewer circulating platelets will be available to interact with the infected RBCs, leading to a less severe form of the disease [35]. However, this assumption is not universally supported. For instance, a Senegalese cohort reported that the presence of thrombocytopenia is associated with an increased risk of childhood mortality [40]. In contrast, a Kenyan cohort [35], as well as the present study, did not report mortality despite elevated IL-10 levels and the presence of thrombocytopenia. Collectively, these findings suggest that the interplay between IL-10, IFN-γ and platelet dynamics in severe malaria is complex, and more host and parasite factors are likely involved.

Although our study is the first to investigate the levels of cytokines in children with severe malaria cases in Omdurman, the national capital of Sudan, there are some limitations that should be addressed for a better interpretation. The major limitation of the current study is the sample size, which is relatively small and probably underpowered. Secondly, we measured the cytokines at the time of presentation only and did not reevaluated at discharge or during recovery, so the exact impact of the therapy on the cytokines cannot be deduced. Thirdly, we measured the cytokines in plasma rather than in tissue samples, which might have shown different interesting findings.

## 5. Conclusions

This study showed that severe *P. falciparum* malaria in Sudanese children is immunologically characterized by an intense, concurrent upregulation of both pro-inflammatory IFN-γ and anti-inflammatory IL-10, reflecting a possible adaptive response to severe malaria infection. Pediatricians should manage malaria cases with thrombocytopenia with high priority. Measurements of IL-10 and IFN-γ at presentation might help differentiate SM from UC cases. Future studies of a prospective nature, including broader multiplex cytokine arrays and multiple time points, including discharge time, is needed.

## Figures and Tables

**Table 1:** Demographic, anthropometric and clinical characteristics of the study participants.

Characteristics	Severe Malaria (SM) (n = 35)	Uncomplicated Malaria (UM) (n = 35)	Healthy Controls (HC) (n = 35)	*p*-Value
**Demographics:**				
**Age, years**	5.0 (2.0–10.0)	6.0 (2.0–9.8)	6.0 (3.5–10.5)	0.730
**Female, n (%)**	17(48.6%)	17(48.6%)	17(48.6%)	>0.99
**Anthropometry:**				
**Weight, kg**	17.0 (10.1–24.5)	20.5 (11.7–32.5)	25.0 (15.5–36.0)	0.231
**Height, cm**	104.0 (86.7–131.0)	116.5 (86.0–134.7)	122.0 (100.0–144.5)	0.219
**Head Circumference, cm**	49.0 (45.5–50.5)	52.0 (48.0–53.0)	53.0 (50.0–54.0)	0.006 *
**Clinical Signs:**				
**Admission Duration (days)**	2.0 (2.0–3.0)	1.0 (1.0–1.0)	N/A	--

Data: Median (IQR) or n (%), N/A = Not applicable, *: significant *p*-value.

**Table 2:** Hematological and biochemical profiles of the study participants.

Laboratory Parameters	Severe Malaria (SM) (n = 35) Median (IQR)	Uncomplicated Malaria (UM) (n = 35) Median (IQR)	Healthy Controls (HC) (n = 35) Median (IQR)	*p*-Value
**Hematology:**				
Hemoglobin (g/dL)	10.5 (7.5–11.6)	11.7 (11.2–12.2)	11.9 (11.3–12.9)	0.001 *
WBC count (×10^3^/μL)	7.5 (4.9–10.1)	6.1 (5.0–7.5)	7.0 (5.2–8.0)	0.302
Platelets (×10^3^/μL)	85.0 (46.5–120.0)	205.5 (145.5–273.1)	290.0 (275.0–359.5)	<0.001 *
Biochemistry:				
Random Glucose (mg/dL)	97.0 (70.0–118.0)	100.0 (94.0–113.0)	94.0 (80.5–100.0)	0.249
Blood Urea (mg/dL)	28.0 (22.0–43.0)	25.5 (20.0–32.0)	22.0 (20.0–27.0)	0.164
Serum Creatinine (mg/dL)	0.5 (0.4–0.7)	0.5 (0.4–0.6)	0.6 (0.5–0.7)	0.711
**Cytokines:**				
Interferon-gamma (IFN-γ), (pg/mL)	848.8 (335.4–1064.9)	219.0 (61.6–966.6)	43.6 (17.7–137.8)	<0.001 *
Interleukin-10 (IL-10), (pg/mL)	292.4 (256.1–315.8)	254.4 (131.9–288.1)	28.1 (19.9–41.1)	<0.001 *

* significant *p*-value.

**Table 3:** Frequency of clinical signs and symptoms of severity and major laboratory abnormalities in children with malaria.

Clinical and Laboratory Features	Severe Malaria (SM) (n = 35)	Uncomplicated Malaria (UM) (n = 35)	*p*-Value
**General Symptoms:**			
Fever (History)	35 (100.0%)	35 (100.0%)	>0.99
Vomiting	35 (100.0%)	32 (91.4%)	0.237
**Clinical Signs of Severity:**			
Pallor	18 (51.4%)	2 (5.7%)	<0.001 *
Respiratory Distress	9 (25.7%)	0 (0.0%)	0.001 *
Heart Failure	8 (22.9%)	0 (0.0%)	0.002 *
Jaundice (Clinical)	6 (17.1%)	0 (0.0%)	0.011 *
Convulsions	3 (8.6%)	0 (0.0%)	0.112
Red Urine (Hemoglobinuria)	2 (5.7%)	0 (0.0%)	0.237
**Laboratory Abnormalities**			
Thrombocytopenia (<164 × 10^3^/μL)	29 (82.9%)	11 (31.4%)	<0.001 *
Severe Anemia (<7 g/dL)	15 (42.9%)	2 (5.7%)	<0.001 *
High Blood Urea (>21 mg/dL)	28 (80.0%)	24 (68.6%)	0.274
Hypoglycemia (RBG < 60 mg/dL)	7 (20.0%)	2 (5.7%)	0.046 *
Leukocytosis (>11 × 10^3^/μL)	8 (22.9%)	3 (8.6%)	0.091
Leukopenia (<4.5 × 10^3^/μL)	6 (17.1%)	4 (11.4%)	0.486
High Creatinine (>1.1 mg/dL)	1 (2.9%)	2 (5.7%)	>0.99

* significant *p*-value.

**Table 4:** Correlation between cytokines and biochemical variables in malaria patients.

Variables	IL-10		Platelets Count		Hemoglobin		Creatinine		Blood Urea		Admission Duration	
	rho	*p*	rho	*p*	rho	*p*	rho	*p*	rho	*p*	rho	*p*
**IFN-γ**	0.688	0.001 *	−0.213	0.105	−0.086	0.516	0.077	0.562	0.154	0.245	0.298	0.022 *
**IL-10**			−0.338	0.009 *	−0.178	0.179	0.063	0.635	0.199	0.130	0.314	0.015 *

* significant *p*-value.

## Data Availability

The data presented in this study are available on request from the corresponding author due to privacy concerns.
